# Inhibition of Nipah Virus Infection In Vivo: Targeting an Early Stage of Paramyxovirus Fusion Activation during Viral Entry

**DOI:** 10.1371/journal.ppat.1001168

**Published:** 2010-10-28

**Authors:** Matteo Porotto, Barry Rockx, Christine C. Yokoyama, Aparna Talekar, Ilaria DeVito, Laura M. Palermo, Jie Liu, Riccardo Cortese, Min Lu, Heinz Feldmann, Antonello Pessi, Anne Moscona

**Affiliations:** 1 Departments of Pediatrics and of Microbiology and Immunology, Weill Medical College of Cornell University, New York, New York, United States of America; 2 Laboratory of Virology, Division of Intramural research, National Institute of Allergy and Infectious Diseases, National Institutes of Health, Hamilton, Montana, United States of America; 3 Department of Biochemistry, Weill Medical College of Cornell University, New York, New York, United States of America; 4 CEINGE, Naples, Italy; 5 PeptiPharma, Rome, Italy; Institut Pasteur, France

## Abstract

In the paramyxovirus cell entry process, receptor binding triggers conformational changes in the fusion protein (F) leading to viral and cellular membrane fusion. Peptides derived from C-terminal heptad repeat (HRC) regions in F have been shown to inhibit fusion by preventing formation of the fusogenic six-helix bundle. We recently showed that the addition of a cholesterol group to HRC peptides active against Nipah virus targets these peptides to the membrane where fusion occurs, dramatically increasing their antiviral effect. In this work, we report that unlike the untagged HRC peptides, which bind to the postulated extended intermediate state bridging the viral and cell membranes, the cholesterol tagged HRC-derived peptides interact with F before the fusion peptide inserts into the target cell membrane, thus capturing an earlier stage in the F-activation process. Furthermore, we show that cholesterol tagging renders these peptides active *in vivo*: the cholesterol-tagged peptides cross the blood brain barrier, and effectively prevent and treat in an established animal model what would otherwise be fatal Nipah virus encephalitis. The *in vivo* efficacy of cholesterol-tagged peptides, and in particular their ability to penetrate the CNS, suggests that they are promising candidates for the prevention or therapy of infection by Nipah and other lethal paramyxoviruses.

## Author Summary

Nipah (NiV) and Hendra (HeV) viruses are two lethal emerging zoonotic paramyxoviruses. In addition to acute infection, these viruses may lead to late-onset disease or relapse of encephalitis years after initial infection, as well as persistent or delayed neurological sequelae. We present a new strategy to prevent and treat NiV/HeV infection that may be broadly applicable for enveloped viral pathogens. Enveloped viruses must fuse their membrane with the target cell membrane in order to initiate infection, and blocking this step can prevent or treat infection, as clinically validated for HIV. For paramyxoviruses, however, peptides that bind the viral fusion protein have been shown to inhibit fusion *in vitro*, but not *in vivo*. The new strategy that we present here opens the door to clinically effective paramyxovirus fusion-inhibitory peptides. By targeting fusion-inhibitory peptides to the target membrane using a cholesterol tag, we capture an early stage in the viral fusion-activation process, thus drastically enhancing the efficacy of these peptides at inhibiting viral entry. Importantly, this strategy prevents and treats lethal Nipah virus infection *in vivo*. Membrane targeting of antiviral peptides thus offers a new approach to development of highly effective peptide fusion antivirals against important human pathogens.

## Introduction

Fusion of enveloped viruses with the host cell is a key step in viral infectivity. The fusion process during viral entry is driven by specialized proteins that undergo an series of conformational changes to bring the viral and host membranes together and promote formation of a fusion pore (reviewed in [1]). For paramyxoviruses — a family that includes a number of important human pathogens — the steps in this process have been explored over the last few years [2], [3], [4], [5], [6], [7], [8], [9], [10], [11], [12], [13]. Each step, from the interaction of the receptor-binding protein with its receptor and the subsequent triggering of the fusion protein, to the conformational changes in the fusion protein that lead to fusion readiness, and the refolding of the fusion protein that drives membrane merger, are potential targets for antiviral compounds. Conversely, the use of compounds that block specific stages of the entry process helps dissect the mechanism of entry.

NiV and Hendra (HeV) viruses are emerging zoonotic paramyxoviruses [14] that are lethal to humans. HeV virus infections currently pose a serious threat to livestock in Australia, where sporadic and deadly transmission to humans has occurred, with the potential for broader dissemination [15], [16], [17]. NiV causes fatal encephalitis in up to 70% of human cases, and causes seasonal outbreaks in Asia [18], [19], with person-to-person transmission now becoming a primary mode of spread [20], [21], [22], [23]. In addition to acute infection, these viruses cause asymptomatic infection in up to 60% of exposed people, and may lead to late-onset disease or relapse of encephalitis years after initial infection [24], as well as persistent or delayed neurological sequelae. No antiviral therapies or vaccines yet exist for HeV or NiV. Other viruses belonging to the paramyxovirus family, particularly respiratory syncytial virus (RSV) and the human parainfluenza viruses types 1, 2, and 3 (HPIV1, 2, 3) [25], [26], cause the majority of childhood croup, bronchiolitis, and pneumonia [27], contributing significantly to global disease and mortality, while measles virus, despite the vaccine, continues to be an important global agent of respiratory and neurologic disease [28].

All paramyxoviruses possess two envelope glycoproteins directly involved in viral entry and pathogenesis: a fusion protein (F) and a receptor-binding protein (HN for the parainfluenza viruses, H for measles, or G for HeV and NiV). The paramyxovirus F proteins belong to the group of “class I” fusion proteins [29], [30], which also includes the HIV-1 gp41 fusion protein. The F protein is synthesized as a precursor protein that is proteolytically processed postranslationally to form a trimer of disulfide-linked heterodimers. This cleavage event places the fusion peptide at the N-terminus of the F1 subunit of the mature F protein, and is essential for membrane fusion activity. For those viruses that fuse with cell membranes at the cell surface, like HPIV, HeV, and NiV, the receptor-binding protein binds to cellular surface receptors, brings the viral envelope in proximity to the plasma membrane, and activates the viral F protein to undergo the required conformational changes leading to fusion. Receptor-ligand interaction is required for the F protein to undergo the series of steps that lead to fusion [7], [9], [31]. It is generally thought that this activation requires prior cleavage of the precursor F.

Peptides derived from the N-terminus heptad repeat (HRN) and the C-terminus heptad repeat (HRC) regions of paramyxovirus fusion (F) proteins can interact with fusion intermediates of F [32], [33], [34], [35], [36], [37]. In particular, peptides from the HRC regions of a number of paramyxoviruses, including Sendai, measles, NDV, RSV, SV5, HeV, and NiV can inhibit the infectivity of the homologous virus [32], [33], [34], [38], [39], [40], [41], [42]. We have shown that peptides derived from the HRC of HPIV3 F are effective inhibitors of both HPIV3 and henipavirus infection [13], [42], and that the efficacy of inhibition depends on both the strength of interaction of the peptide with the target fusion protein and the time window of access to the target sequence. By adding a cholesterol group to an HPIV3 HRC-derived peptide, and thus targeting the peptide to the membrane where fusion occurs, the peptide becomes a highly effective inhibitor [43].

The current paradigm for the mechanism of HR-derived peptide action proposes that HRC peptides bind to the postulated extended intermediate state, after the fusion peptide has inserted into the target membrane, and prevent the transition to the post-fusion conformation [5], [44], [45]. Accordingly, peptides with greater affinity for the intermediate state are better inhibitors. This has led us (see below) and others to use structure-based design to improve inhibitor potency. However, once fusion has proceeded past the extended intermediate stage of F, peptide inhibitors are ineffective. We have therefore proposed that the design of more effective HRC peptide inhibitors should also aim at enhancing access and association of inhibitor with the intermediate-stage fusion protein, either by increasing the concentration of inhibitor at the location of receptor binding, or by targeting an earlier stage of F-activation [13]. Here, we report that the cholesterol-tagged HRC-derived peptides interact with the F protein before the fusion peptide inserts into the target membrane in the extended intermediate state, thereby targeting and capturing an earlier stage in the F-activation process than do the corresponding untagged peptides. The stage of F-activation that is targeted by these peptides is dependent upon HN-receptor interaction, but occurs prior to the insertion of the fusion peptide into the target cell membrane. These findings contrast with the existing paradigm that HR-derived peptides cannot bind to F before F activation and insertion into the target membrane [13], [45], [46], [47]. In agreement with our hypothesis, these peptides are potent antiviral compounds *in vivo*, that accumulate in the CNS and effectively treat NiV encephalitis in an established animal model.

## Results

### Design of improved HRC inhibitors

We addressed the design of improved HPIV3/NiV inhibitors in four steps: (i) analysis of the crystal structure of the heterotypic 6HB complex; (ii) design of mutations aimed at improving interhelical interaction; (iii) biophysical studies to prove that the designed mutations yield 6HB with increased thermodynamic stability, and (iv) combine the stability-enhancing mutations with cholesterol conjugation. While steps (i)-(iii) were aimed at decreasing the k_off_ of the inhibitor-F complex, step (iv) was aimed at increasing the k_on_ for complex formation, and the two strategies were expected to be additive, if not synergistic.

**Figure 1:**
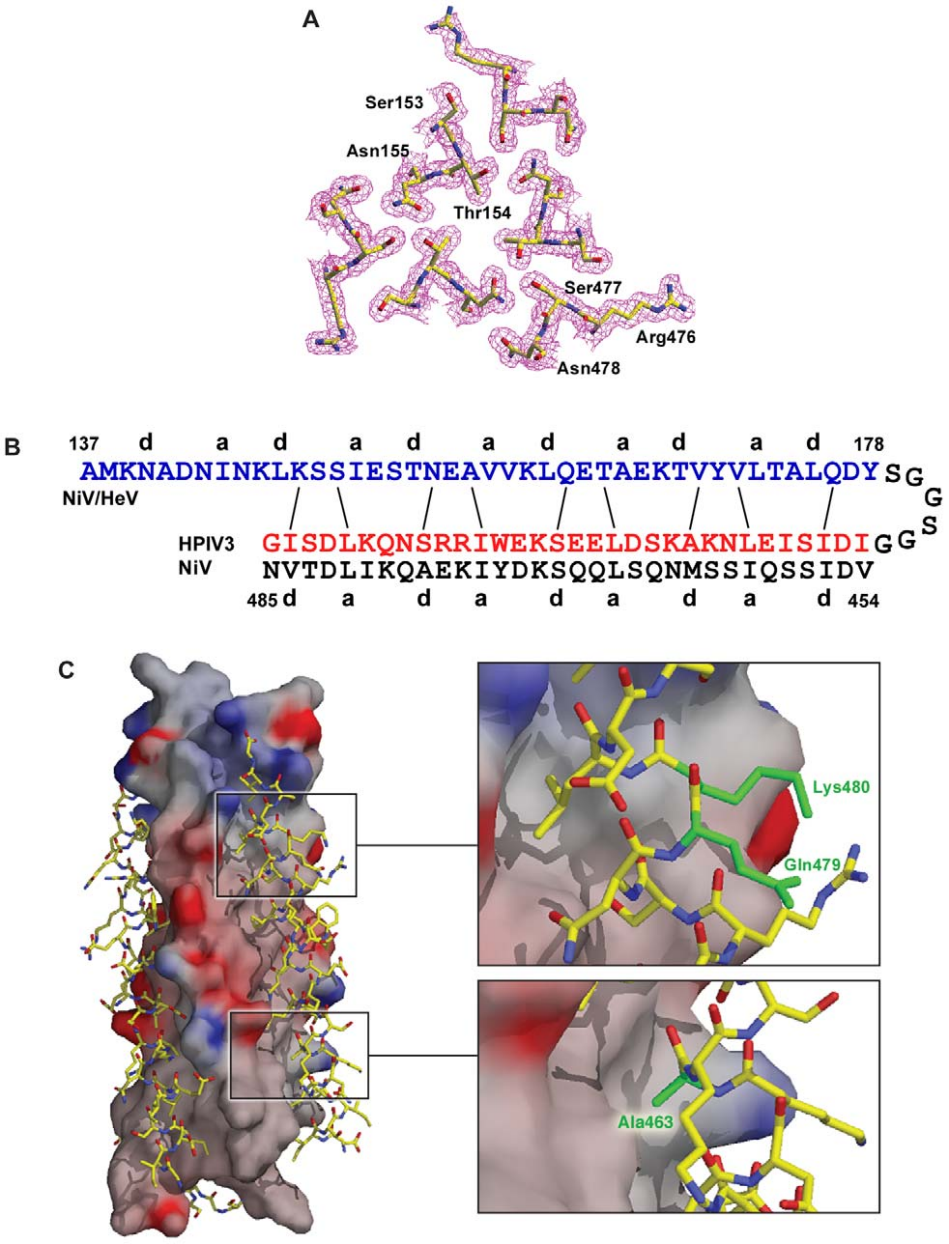
Crystal structure of the chimeric six-helix bundle formed by the NiV/HeV HRN segment N42 and the HPIV3 HRC segment C32. (A) Interacting cross-sectional layer of the N42_NiV_(L6)C32_HPIV3_ trimer. The 2*F*_o_ – *F*_c_ electron density map (contoured at 1.0sigma) is shown with the refined molecular model. (B) Schematic representation of interfacial interactions between two adjacent N42_NiV_ helices and a C32_HPIV3_ helix. Packing interactions between residues at positions ***e*** and ***g*** of N42_NiV_ and residues at positions ***d*** and ***a*** of C32_HPIV3_ are indicated by solid lines. (C) Surface interaction properties of the N42_NiV_ triple-stranded coiled coil with three C32_HPIV3_ chains drawn as an atomic model. The solvent-accessible surface is colored according to the local electrostatic potential, ranging from +13 V in dark blue (most positive) to –10.3 V in deep red (most negative). The positions of Ala463, Gln479 and Lys480 (colored in green) in C32_HPIV3_ are shown.

Crystal structure of the chimeric N42_NiV/HeV_(L6)C32_HPIV3_ complex: We previously showed that peptides corresponding to the HRC region of human parainfluenza virus type 3 (HPIV3) are better inhibitors of the heterologous paramyxoviruses Nipah (NiV) or Hendra (HeV) than those derived from the HRC regions of NiV or HeV themselves [42], [48]. HPIV3 HRC peptides act by binding to the F HRN coiled coil, disrupting postfusion six-helix bundle (6HB) formation and blocking viral entry. The basis for this phenomenon lies in the greater stability of the interaction between the heterologous HRN-HRC pair (NiV-HPIV3) compared to the homologous pair (NiV-NiV) [48]. To explain the greater stability of the heterologous pair, we expanded on our previous residue-by residue evaluation of the HRN-HRC interaction, and studied this interaction in atomic detail. Based on the published crystal structures of the HeV and NiV F six-helix bundles [49], we designed and constructed a soluble polypeptide corresponding to the chimeric fusion core (called N42_NiV/HeV_(L6)C32_HPIV3_), and consisting of the NiV/HeV HRN N42 and HPIV3 C32 peptides connected *via* a short peptide linker (Figure 1B); we deleted the first proline residue in the HPIV3 HRC sequence in order to facilitate crystallographic studies.

The crystal structure of N42_NiV/HeV_(L6)C32_HPIV3_ was solved by molecular replacement (Table 1). The final 1.80-Å 2*F*_o_ – *F*_c_ electron density map reveals the positions of all the amino-acid residues except 19 at the helix termini and in the linker region (Figure 1A). The quality of the structure was verified with simulated annealing omit maps and by PROCHECK [50]. All protein residues occupy the most favored regions of the Ramachandran plot.

**Table 1:** Crystallographic data and refinement statistics.

**Data collection**	
Resolution (Å)	67.1–1.80
No. of unique reflections	20,844
Redundancy	5.4 (5.1)*
Completeness (%)	92.5 (75.3)
*R*_merge_ (%)	5.9
*I*/*s* *I*)	13.7 (2.6)
Space group	*P*2_1_2_1_2_1_
Unit-cell parameter	a = 32.4 Å, b = 54.1 Å, c = 134.1 Å
No. of molecules in AU	3
Solvent content (%)	43.5
**Refinement**	
Resolution (Å)	67.1–1.80
No. of reflections	20,844
No. of protein atoms	1,738
No. of water molecules	156
No. of citrate ion	1
No. of t-butanol	2
*R*_cryst_/*R*_free_ (%)	19.4/24.2
Average *B*-factor (Å^2^)	32.2
*rms* deviations	
Bond lengths (Å)	0.011
Bond angles (°)	1.2
*B* values (Å^2^)	5.8

*The highest resolution shell (1.86–1.80 Å) is shown in parenthesis.

Like the wild-type NiV/HeV F core structure, the chimeric N42_NiV/HeV_(L6)C32_HPIV3_ construct folds into a six-helix bundle composed of three helical hairpins, each consisting of an N42 helix paired with an antiparallel C32 helix (Figure 1). At the center of the bundle, the side chains at the heptad ***a*** and ***d*** positions of the NiV/HeV N42 coiled coil displayed typical “knobs-into-holes” packing interactions (Figure 1A). Three HPIV3 C32 helices wrap in the reverse direction against the outside of the N42 coiled coil. These C32 helices interact with the N42 helices through residues in three highly conserved hydrophobic grooves on the surface of the coiled-coil trimer (Figure 1C). In general, residues in the ***a*** and ***d*** positions of the HPIV3 C32 helix pack against residues at the ***e*** and ***g*** positions of the NiV/HeV N42 helices, while the peripheral ***e*** and ***g*** side chains of C32 also participate in the hydrophobic interactions (Figure 1B). The root mean square (rms) deviation between all corresponding C atoms of the central N42 coiled coil in the wild-type and chimeric six-helix bundles is 1.28 Å. The corresponding C atoms of the C32 helices in N42_NiV/HeV_L6)C32_HPIV3_ can also be superimposed upon the wild-type NiV/HeV counterpart with a rms deviation of 1.86 Å. Thus, the overall architecture and helix packing of the chimeric construct are the same as that of the wild-type NiV/HeV F Core.

Although most of the helix-packing interface within the F core structure involves hydrophobic interactions, interhelical hydrogen bonds and salt bridges are uniformly distributed along the hydrophobic contacts. In the chimeric structure, a cluster of polar residues, including Ser153, Thr154, Asn155 of HeV/NiV N42, and Arg476, Ser477, Asn478 of HPIV3 C32 (Figure 1A), is involved in an intricate network of interhelical hydrogen bonds. These favorable van der Waals and polar interactions in the chimeric 6HB impart strong helical character and binding energy to the HPIV3 HRC peptide for binding to the trimeric coiled coil of HeV/NiV F, as predicted by a dominant-negative mode of inhibition. Our results provide strong evidence that interhelical packing interactions in the F ectodomain core are important determinants of the stability of HRN-HRC interaction, and thus of peptide inhibitor activity.

Design of peptides with improved interhelical packing: Based on the above data, we sought to identify amino acid changes that would increase heterotypic binding of the inhibitor to the HeV/NiV coiled coil, while not perturbing, or possibly improving, its homotypic binding to the HPIV3 coiled coil. First, the crystal structures of the homo- and heterotypic six-helix bundles revealed that HPIV3 Ala463 at a ***d*** position packs against Val169 of the NiV coiled coil, while the corresponding NiV Met463 side chain makes prominent contacts with hydrophobic grooves on the surface of the coiled coil. Therefore in the HPIV3 HRC peptide inhibitor, we substituted Ala463 with isoleucine, a preferred residue in the ***d*** position and not prone to oxidation as methionine, to strengthen its packing interactions with both HPIV3 and NiV/HeV F.

Second, we considered the two residues at position 479–480, Gln479-Lys480 in HPIV3 and Lys479-Ile480 in HeV/NiV, and reasoned that introducing the 479–480 HeV/NiV residues in the inhibitor to replace the corresponding HPIV3 residues would result in several advantages: (i) The NiV Ile480 residue at a ***g*** position is involved in the interhelix interaction with NiV Ser150, while Lys480 in HPIV3 is not involved in a similar interaction. Therefore substitution of Lys480 (HPIV3) with Ile480 (NiV) would provide improved binding to the HeV/NiV coiled coil, and might simultaneously improve binding to the HPIV3 coiled coil by maintaining the hydrophobic packing of the methylene groups of Lys480, while removing its buried charged and polar atoms; (ii) NiV Lys479 at a ***f*** position forms an intra-helical hydrogen bond with NiV Glu476, but this H-bond is not observed between the HPIV3 Gln479 and Arg476 residues. Substitution of Gln479 (HPIV3) with Lys479 (NiV) therefore would not cause loss of an interaction, while it would enhance helicity of the inhibitor. This should increase the binding affinity for both HPIV3 and HeV/NiV by decreasing the entropy penalty for folding; (iii) Exchanging the HPIV3 Gln-Lys pair with the NiV Lys-Ile pair would not change the local electrostatic balance.

Biophysical studies of mutated 6HBs: To directly test whether these mutations enhance stability of the 6HB, we mutated the Ala463, Gln479 and Lys480 residues of the C32 segment in the recombinant N42(L6)C32 model of the homotypic and chimeric F 6HBs [48]. We compared the stability of the 6HBs formed between HRN-peptides (both HPIV3 and NiV) and the previously described HRC peptides [48] with the 6HBs formed with the new VIKI mutant (containing the mutations E459V, A463I, Q479K, K480I). Note that all peptides bear the Glu459-to-Val mutation that we previously identified as enhancing HRN-HRC interaction and potency [48].

**Figure 2:**
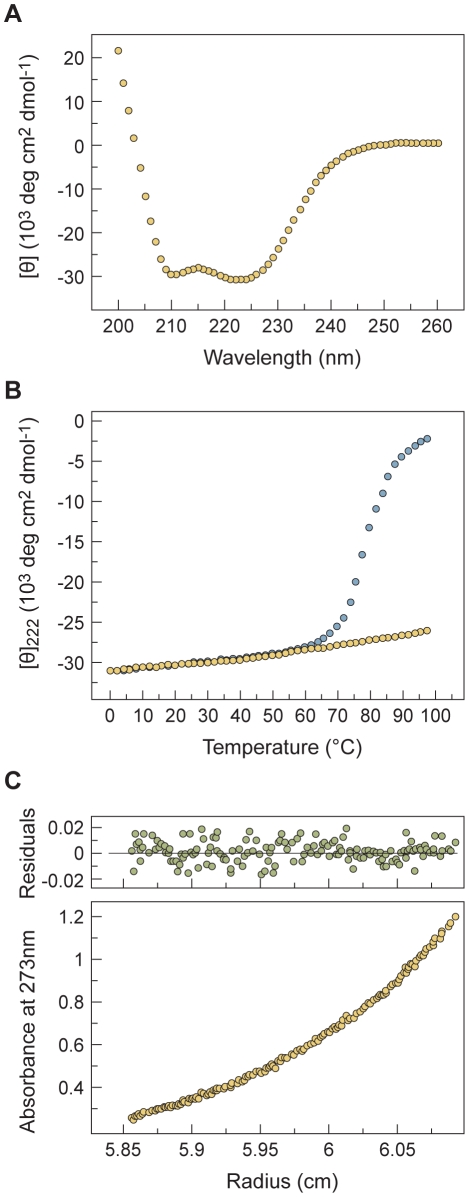
Engineered mutations enhance the interactions of two hydrophobic heptad-repeat (HRN and HRC) regions in NiV and HPIV3 F. Ala463-to-Ile, Gln479-to-Lys and Lys480-to-Ile mutations (in the HPIV3 F HRC region) stabilize the heterotypic six-helix bundle structure formed by the recombinant N42_NiV_(L6)C32_HPIV3-V_ model. (A) Circular dichroism (CD) spectrum of N42_NiV_(L6)C32_HPIV3-VIKI_ (50 uM) in TBS (pH 8.0) at 4°C. (B) Thermal melts of N42_NiV_(L6)C32_HPIV3-VIKI_ monitored by CD at 222 nm at 50 uM protein concentration in TBS (pH 8.0) in the presence (open circles) and absence (filled circles) of 3 M GuHCl, a chemical denaturant. (C) Representative sedimentation equilibrium data (19 krpm) of N42_NiV_(L6)C32_HPIV3-VIKI_ collected at 20°C in TBS (pH 8.0) at ∼30 uM protein concentration. The natural logarithm of the absorbance at 273 nm is plotted against the square of the radial position. The random distributions of the residuals indicate that the data fit well to an ideal single-species model. The slope of the plotted data indicates that N42_NiV_(L6)C32_HPIV3-VIKI_ is a trimer.

Circular dichroism (CD) spectroscopy indicated that the recombinant proteins (N42_HPIV3_(L6)C32_HPIV3-VIKI,_ and N42_NiV_(L6)C32_HPIV3-VIKI_) were well folded and contained >90% helical structure at 4°C in 50 mM Tris-HCl (pH 8.0) and 150 mM NaCl (TBS) (Table 2, Figure 2A). Analytical ultracentrifugation experiments indicated that each protein forms a discrete trimer over a concentration range from 30 to 300 uM (Table 2, Figure 2C). The thermal stability of the homotypic and chimeric N42(L6)C32 6HB for the panel of different peptides was assessed using CD by monitoring the ellipticity at 222 nm as a function of temperature at 50 µM protein concentration in TBS (pH 8.0). N42_HPIV3_(L6)C32_HPIV3-VIKI,_ and N42_NiV_(L6)C32_HPIV3-VIKI_ have thermal stabilities that exceed 100°C (Table 2, Figure 2B). In the presence of the denaturant guanidine hydrochloride (GuHCl) at 3 M concentration, N42_NiV_(L6)C32_HPIV3-VIKI_ peptides unfold cooperatively, with an apparent melting temperature (*T*_m_) of 78°C (Figure 2B), compared to a *T*_m_ of 72°C for the parent N42_NiV_(L6)C32_HPIV3-V_ with the Glu459-to-Val mutation and to a *T*_m_ of 67°C for the original HPIV3 sequence N42_NiV_(L6)C32_HPIV3_ (Table 2, [48]). On the other hand, N42_HPIV3_(L6)C32_HPIV3-V_ forms an unusually stable helical structure, with an apparent *T*_m_ of >95°C in the presence of 5 M GuHCl [48] even without the Ile-Lys-Ile mutations, and N42_HPIV3_(L6)C32_HPIV3-VIKI_ has an apparent *T*_m_ of >100°C under the same conditions [48] (Table 2). Taken together, these results indicate that the Ile-Lys-Ile substitutions in the VIKI mutant yield a more stable interaction with both (NiV and HPIV3) HRNs compared to the previously described peptide.

**Table 2:** Biophysical data of mutant N42(L6)C32 polypeptides.

	–[*q*]_222 nm_ (deg cm^2^ dmol^–1^)	*T*_m_ (°C)	*T*_m_^3M^ ^GuHCl^ * (°C)	*T*_m_^5M^ ^GuHCl^ * (°C)	M_obs_/M_calc_ 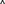
N42_NiV_(L6)C32_HPIV3-V_	34,600	>100	72	<0	2.9
N42_NiV_(L6)C32_HPIV3-V/IKI_	32,000	>100	78	<0	3.0
N42_HPIV3_(L6)C32_HPIV3-V_	32,600	>100	>100	>95	3.0
N42_HPIV3_(L6)C32_HPIV3-V/IKI_	32,800	>100	>100	>100	3.1

*T_m_^GuHCl^ denotes the apparent melting temperature in 3 or 5 M GuHCl. All CD scans and melts were performed on 50 mM protein solutions in TBS (pH 8.0).

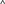
M_obs_/M_calc_ is the apparent molecular mass determined from sedimentation equilibrium data divided by the calculated molecular mass of a monomer.

Combining stability-enhancing mutations with cholesterol conjugation: We have previously shown that addition of a cholesterol moiety to the C-terminus of a 36-amino acid peptide corresponding to the HRC region of HPIV3 F (V-chol, Table 3) improves the antiviral activity by 2 logs [43]. The cholesterol conjugation increases the concentration of the inhibitor in cholesterol-rich lipid rafts, where viral fusion is triggered after the initial attachment of the virus to the host cell [43], [51], [52]. We conjugated a cholesterol group to the VIKI mutant. In doing so, we also introduced a small spacer made of 4 units of polyethylene glycol (PEG_4_), a well-known solubilizing moiety, between the peptide and the cholesterol moiety, to improve the physicochemical properties of the conjugated peptide (VIKI-PEG_4_-chol, Table 3). We reasoned that a flexible spacer between the membrane-bound cholesterol moiety and the cysteine residue to which it is attached might also reduce any steric strain resulting from the need of simultaneous, oriented binding of the inhibitor to the membrane and the HRN moiety of F – the relative geometry of which is expected to change during the progress of fusion. Accordingly, we found that the PEG spacer not only considerably improves peptide solubility in acqueous media, but also enhances activity.

**Table 3:** Comparison of properties of cholesterol-tagged peptides.

Short name	PEG linker	HPIV3 F derived HRC sequence	IC50 (nM)			
			NiV		HPIV3	
			infection*	fusion	infection	fusion
V-chol	NO	449-VALDPIDISIVLNKAKSDLEESKEWIRRSNQKLDSI-484	∼0.02	∼300	∼10.0	>1000
V-PEG_4_-chol	YES	449-VALDPIDISIVLNKAKSDLEESKEWIRRSNQKLDSI-484	∼0.02	∼80.0	∼0.70	>1000
VIKI-PEG_4_-chol	YES	449-VALDPIDISIVLNK *I* KSDLEESKEWIRRSN*K I* LDSI-484	∼0.02	∼5.0	∼0.30	∼30.0

*PSEUDOTYPE ASSAY.

### The sequence-optimized, cholesterol-tagged peptide is the most potent inhibitor of HPIV3 infectivity

**Figure 3:**
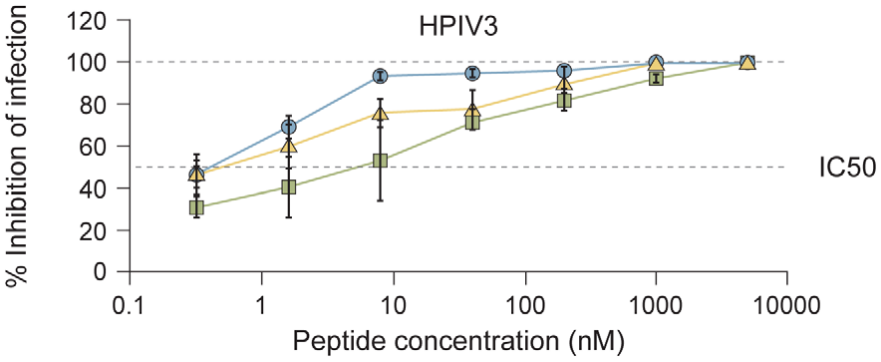
Inhibition of HPIV3 infection by HPIV3 F HRC cholesterol-tagged peptides. CV1 cell monolayers were infected with wild type HPIV3 at a multiplicity of infection (m.o.i.) of 6.7×10^−4^ in the presence of increasing concentrations of the VIKI-PEG_4_-chol (circle), V-PEG_4_-chol (triangle) or V-chol (square) peptides. After a 90-min incubation at 37°C, cells were overlaid with methylcellulose, and plaques were stained and counted after 24 h. The percent inhibition of viral entry (compared to results for control cells infected in the absence of inhibitors) is shown as a function of the (log-scale) concentration of peptide. Data points are means (± SD) for triplicate samples. These data are representative of results from three to five experiments.

The inhibitory activity of V-chol, V-PEG_4_-chol, and VIKI-PEG_4_-chol (Table 3) against HPIV3 was assessed in a plaque reduction assay (Figure 3). The peptides containing the PEG_4_ spacer both performed better than the original peptide. While for the cholesterol-tagged peptide with no spacer the IC_50_ value is ∼10 nM, for PEG_4_-containing peptides the IC_50_ value decreases to ∼0.7 nM (V-PEG_4_-chol) and ∼0.3 nM (VIKI-PEG_4_-chol). The VIKI peptide outperformed the other peptides over a wide range of concentrations. Taken together, the data in Figure 3 indicate that (i) the effects of cholesterol and of the amino acid substitutions are additive, and (ii) addition of a PEG spacer between the C-terminal cysteine and the cholesterol group independently and further improves the overall efficacy of the entry inhibitor against HPIV3.

### The sequence-optimized, cholesterol-tagged peptide inhibits viral infection in human airway epithelium

For clinical utility, the ability of an antiviral agent to prevent multiple rounds of infection is key. We recently established that a human airway epithelium (HAE) model closely reflects the *in vivo* behavior of a panel of HPIV3 variants [53]. The HAE model was previously used to characterize the cell specificity of respiratory syncytial virus [54] and HPIV3 [55], a study which confirmed that the model replicates those paramyxovirus-HAE interactions that occur in the human lung. We also recently employed this system to test a sialidase-based inhibitor, and showed a direct correlation between the HAE and *in vivo* results [56]. We therefore hypothesized that peptide efficacy in this *ex vivo* model would correlate with *in vivo* efficacy.

**Figure 4:**
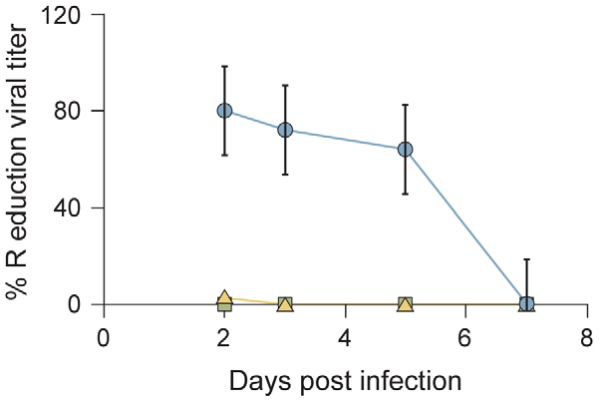
VIKI-PEG_4_-chol peptide efficiently curtails multicycle replication in HAE culture. HAE were infected with HPIV3 in the presence of 1uM VIKI-PEG_4_-chol (circle), V-PEG_4_-chol (triangle) or V-chol (square) peptide. After a 90 min incubation at 37°C, the inoculum was removed and the HAE were incubated at 37°C. The virus released from the apical surface was collected at the indicated time points. The percent reduction in viral titer (compared to results from mock infected samples) is shown as a function of time. Values represent means ± SD of 3 replicate samples. These data are representative of an experiment repeated 3 times.

We assessed the effect of the cholesterol tagged peptides on viral infection in HAE (Figure 4). The pseudostratified epithelium was infected at the apical surface with wt HPIV3 in the presence of 1000 nM of each peptide. After a 90 minute adsorption period, the liquid from the apical surface, containing virus and peptide, was aspirated, and the titer of virus emerging from the apical surface was measured at 2, 3, 5, and 7 days post-infection [53]. Figure 4 shows that at two days post-infection, neither the V-chol nor the V-PEG_4_-chol peptide reduced the viral titer; the titer in the presence of those peptides is the same as for untreated control wells. At the same time point, after a single initial dose of the VIKI-PEG_4_-chol peptide, the titer was reduced by 80%. Even at 5 days after infection, the single initial dose of VIKI-PEG_4_-chol peptide reduced viral titer by ∼50%. Thus, a single dose of the VIKI-PEG_4_-chol peptide blunted the viral titer in HAE over several days; only after 7 days we observe a major decline in the inhibitory effect. The viral titers at each time point are presented in table S1. These results provided support for the efficacy of tagged peptides in the natural host and will lead to future experiments in an animal model.

### The sequence-optimized, cholesterol-tagged peptide is the most potent inhibitor of both HPIV3 and NiV fusion

**Figure 5:**
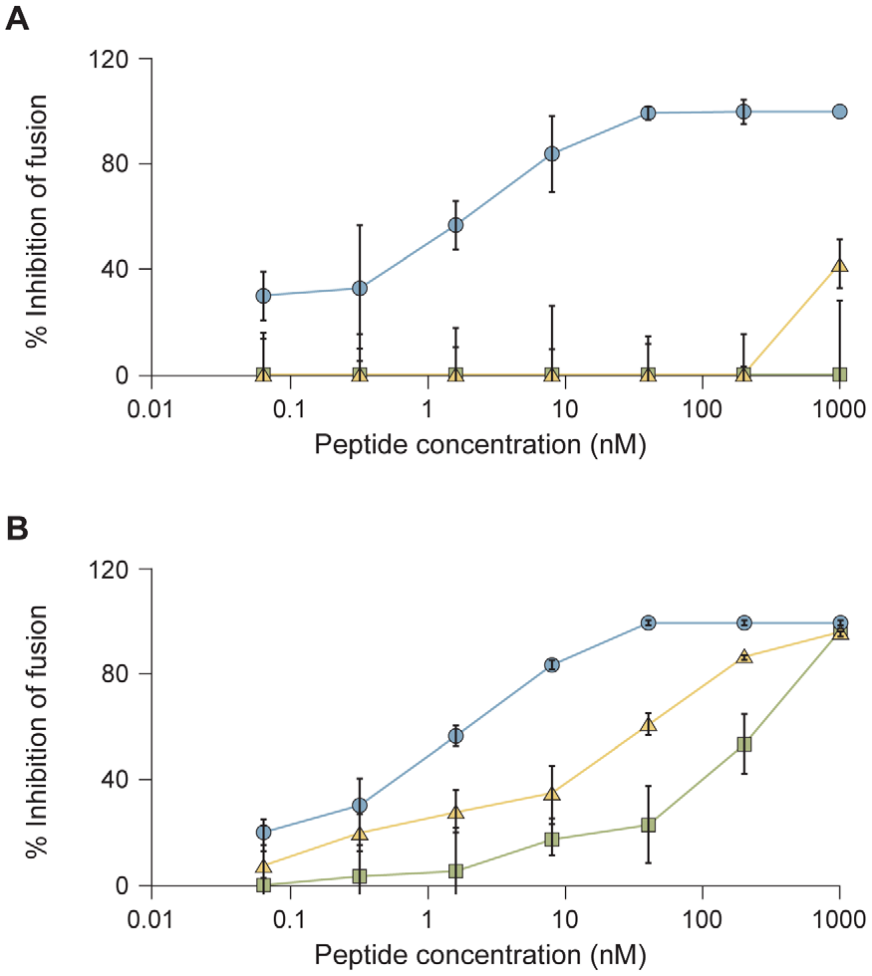
HPIV3 F HRC cholesterol-tagged peptides inhibit cell fusion mediated by HPIV3 HN/F and NiV G/F. HPIV3 HN/F (A) or NiV G/F (B) co-expressing cells were allowed to fuse with sialic acid receptor-bearing cells (293T) in the presence of increasing concentrations of VIKI-PEG_4_-chol (circle), V-PEG_4_-chol (triangle) or V-chol (square) peptide. After 4 hours, the amount of fusion was quantitated using a beta-galactosidase complementation assay as described in Materials and Methods. The percent inhibition of fusion (compared to results for control cells not treated with peptide) is shown as a function of the (log-scale) concentration of peptide. The values are means (± SD) of results from three experiments.

While in HAE the VIKI-PEG_4_-chol peptide inhibition of viral titer was far superior to the other two cholesterol-tagged peptides (Figure 4), all three peptides completely inhibited infection in monolayer culture (Figure 3) at the concentration of peptide that was used in the HAE (1000 nM). To address this difference, we turned to a quantitative fusion assay based on beta-galactosidase complementation, that we have found to accurately reflect the HAE and in vivo results in the assessment of inhibitors [12], [42], [53]. For the experiment shown in Figure 5, fusion mediated by HPIV3 or NIV in the presence or absence of inhibitory peptides was measured after incubation at 37°C for 4 hours. The peptides were present during the entire fusion process, allowing them to act at the stages of triggering/activation of the fusion protein, or during subsequent fusion.

Figure 5 shows the percent inhibition of fusion mediated by either HPIV3 F coexpressed with HPIV3 HN (Figure 5A) or NiV F coexpressed with NiV G (Figure 5B), in the presence of increasing concentrations of V-chol peptide (squares), V-PEG_4_-chol peptide (triangles) or VIKI-PEG_4_-chol peptide (circles). Fusion between cells expressing viral envelope glycoproteins (HPIV3 HN/F or NiV G/F) and the alpha beta-gal subunit and cells expressing the omega beta-gal subunit was quantitated. Fusion mediated by the HPIV3 glycoproteins F and HN was inhibited only by the VIKI-PEG_4_-chol peptide, with an IC_50_ value of ∼30 nM and maximal inhibition at 50 nM (Figure 5A). The other two peptides did not inhibit fusion even at the highest concentration used here (1000 nM).

While cell fusion mediated by NiV F coexpressed NiV G was inhibited by all three peptides, at each concentration, the VIKI-PEG_4_-chol peptide was more inhibitory than the other two peptides (Figure 5B). These results for both HPIV3 and NiV led us to further explore the basis for the VIKI-PEG_4_-chol peptide's efficacy.

### The sequence-optimized, cholesterol-tagged peptide interacts with F prior to the fusion peptide insertion into the target membrane

We hypothesized that the IKI mutations in the VIKI-PEG_4_-chol peptide might affect the peptide's mechanism of action. To investigate the point in the fusion process at which VIKI-PEG_4_-chol acts, we used an assay that we previously designed to distinguish between different states of F activation [7], [12]. This assay allowed us to address that the VIKI-PEG_4_-chol and the other two peptides target different states of F activation.

To determine whether the peptides interact with F before insertion of the fusion peptide into the cell membrane, we first used a cleavage site mutant F (F-csm) that does not expose the fusion peptide, and therefore cannot mediate membrane merger [11], [57], and an HPIV3 HN (T193A HN) that possesses high binding avidity and therefore dissociates from its receptor only in the presence of zanamivir [12]. The F-csm permits assessment of the stages the fusion sequence prior to fusion peptide membrane insertion, and the T193A HN removes the variable of receptor dissociation during the experiment, allowing us to focus on potential interactions of peptide with F prior to membrane insertion.

**Figure 6:**
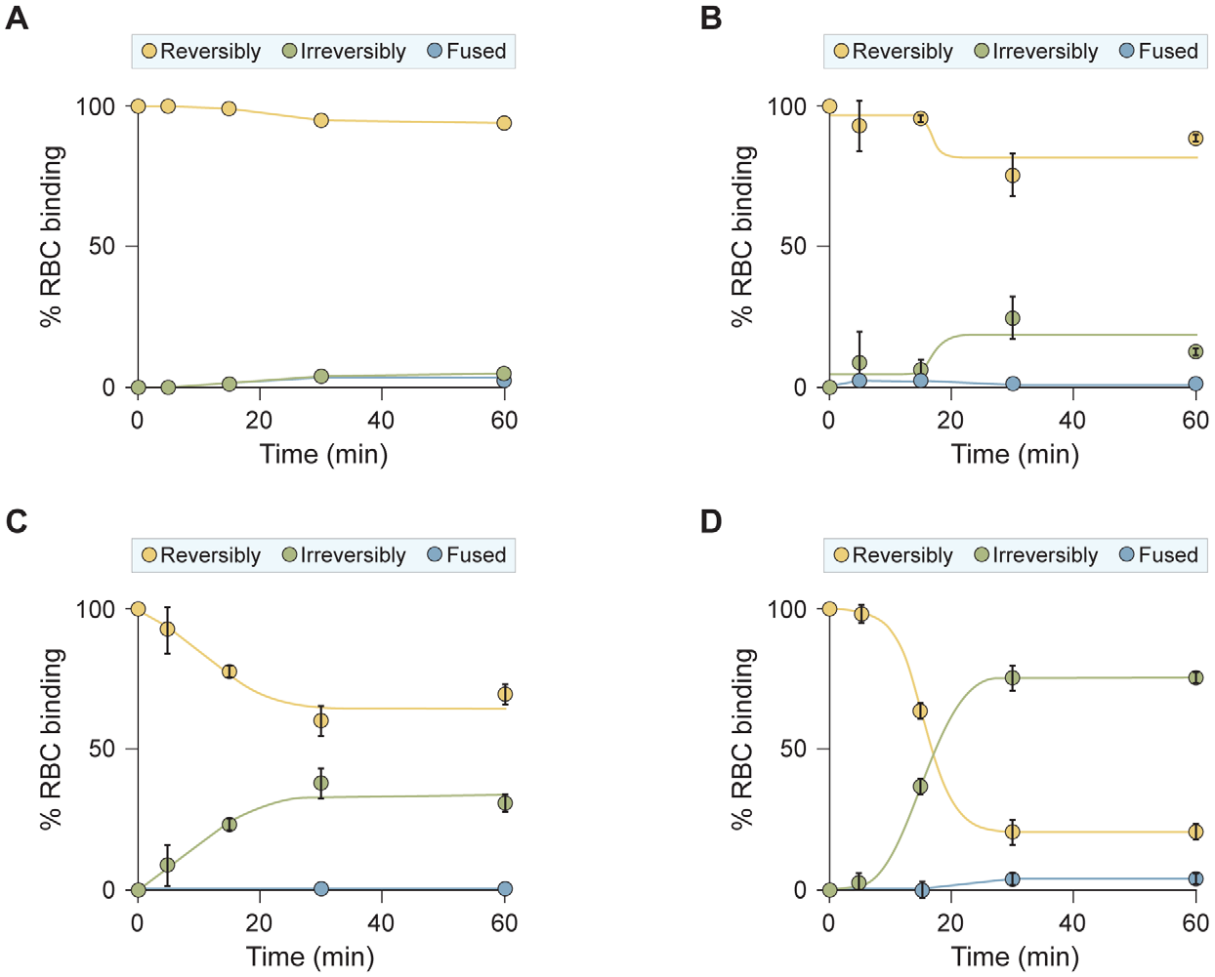
HPIV3 F HRC cholesterol-tagged peptides interact with uncleaved F: peptide-F interaction attaches HN/F-expressing cells to receptor-bearing target cells. Monolayers of cells co-expressing HN and uncleaved (cleavage site mutant, csm) F were allowed to bind to receptor-bearing RBCs at 4°C. Upon transfer to 37°C, media containing (A) no peptide, (B) 1 uM V-chol peptide, (C) 1 uM V-PEG_4_-chol peptide, or (D) 1 uM VIKI -PEG_4_-chol peptide were added. Zanamivir was added to block HN-receptor interaction at the indicated timepoints and RBCs that were reversibly bound by HN-receptor interaction (yellow), irreversibly bound (green), or fused (blue) were quantified. The ordinate values are means (± SD) of results from triplicate samples.

To compare the stage of peptide action between the VIKI-PEG_4_-chol and the other two peptides (Figure 6), cells co-expressing HPIV3 F-csm and T193A HPIV3 HN were allowed to bind erythrocytes (RBCs) at 4°C. The cells were then washed and medium containing V-chol, V-PEG_4_-chol, VIKI-PEG_4_-chol (at a concentration of 1 uM), or no peptide, was added. The cells were transferred to 37°C to allow F-activation to occur, and at the indicated time points zanamivir was added to interfere with HN-receptor interaction [7], [8], [9], [10], [58], [59]. We determined the amount of target RBCs that (a) were released into the medium by zanamivir addition (yellow), (b) were bound but had not fused (green), or (c) had undergone fusion (blue). As expected, no fusion was mediated by F-csm. In the absence of peptide (Figure 6A) all of the RBCs were released by zanamivir, indicating that binding is completely HN-dependent at all the time points. However, for all the cholesterol tagged peptides, there is a progressive increase in the quantity of RBCs that are not detached by zanamivir, and are thus irreversibly bound. This result indicates that in the presence of the cholesterol-tagged peptides, RBCs are retained in an HN-independent manner, suggesting that the peptide-F interaction causes the RBCs to remain attached to the HN/F-expressing cells. This effect is observed only with the C-terminally cholesterol-tagged peptides; N-terminally tagged or untagged peptides show no RBC retention (Figure S1).

Two potential interpretations of these findings are that: (1) the cholesterol-tagged peptides, which are anchored in the target RBC membrane, interact with the uncleaved F molecule and form a bridge between the HN/F-expressing target cell membrane and the RBC membrane, or (2) the peptide is interacting with the uncleaved F molecule and thereby stabilizing F binding to an unknown “F receptor” [46]. We disfavor this second possibility since we find that only insertion of peptide into the RBC (target) cell membrane, and not into the HN/F-expressing membrane, leads to the peptide-F interaction (Figure S2).

**Figure 7:**
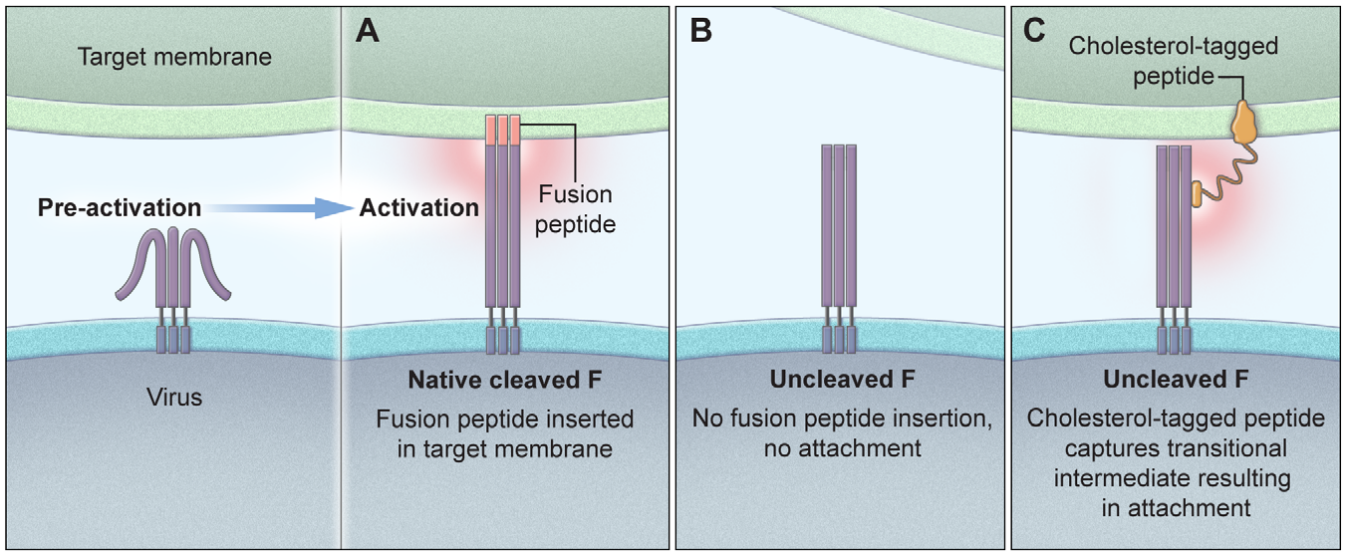
Schematic illustration of the proposed mechanism of cholesterol-tagged peptides. The panels show the interaction of cholesterol-tagged peptides with the uncleaved F molecule (B) and the formation of a bridge between the HN/F-expressing cell and the target membrane (C). For comparison, native cleaved F is shown with the fusion peptide inserted into the target cell (A).

The diagram in Figure 7 shows our proposed mechanism for the interaction of cholesterol-tagged peptides with the uncleaved F molecule and the formation of a bridge between the HN/F-expressing cell and the target membrane. Panel A schematically represents native, cleaved F after activation and insertion of the fusion peptides into the target membrane. In panel B, F is uncleaved and there is no fusion peptide insertion and therefore no irreversible attachment to the target membrane. In panel C, the uncleaved F is schematically represented as attached to the target membrane via the cholesterol-tagged peptide's interaction with both the target membrane and the uncleaved F. We postulate that for this to occur, the uncleaved F must have undergone conformational change to expose the HRN regions, and the cholesterol-tagged peptide then captures this transitional intermediate.

While all three cholesterol peptides mediate HN-independent attachment, there are clear differences between them: VIKI-PEG_4_-chol promotes rapid irreversible binding, V-chol is the slowest to promote irreversible binding (Figure 6B) and V-PEG_4_-chol achieved irreversible retention in an intermediate time (Figure 6C). Importantly, the ability of each peptide to promote RBC retention is well correlated with the inhibitory efficacy of each peptide (Figure 4 and 5).

**Figure 8:**
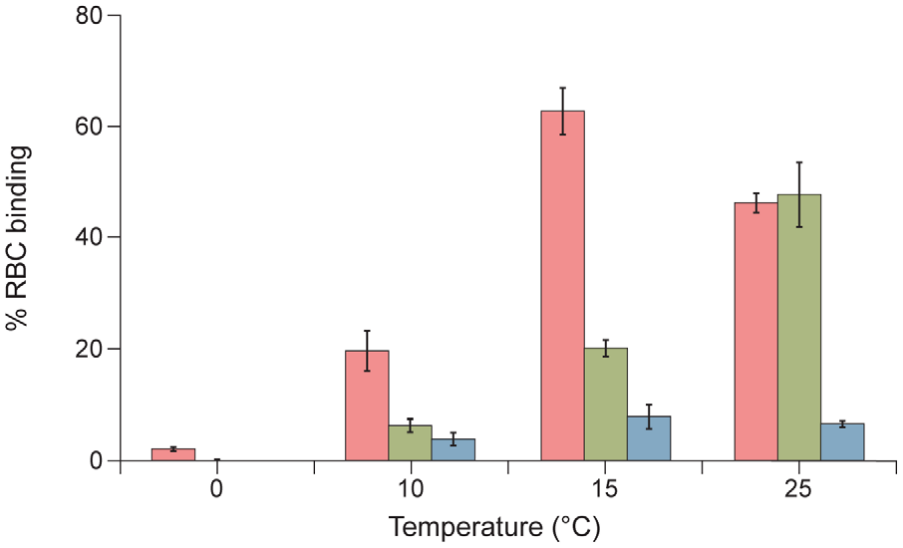
HPIV3 F HRC cholesterol-tagged peptides interact with native cleaved F: peptide-F interaction attaches HN/F-expressing cells to receptor-bearing target cells at an early stage of F-activation. Monolayers of cells co-expressing HN and F were allowed to bind to receptor-bearing RBCs at 4°C. Upon transfer to the indicated temperatures, media containing no peptide (blue bar), 10 uM VIKI -PEG_4_-chol (red bar), or 10 uM VIKI-untagged peptide (green bar) were added. At 60 minutes, zanamivir was added to block HN-receptor interaction and RBCs that were irreversibly bound were quantified. The values are means (± SD) of results from triplicate samples.

To confirm that the results obtained with F-csm are valid also for wild-type F, we repeated the assay described in Figure 6 using native F and a range of temperatures, including temperatures (0^o^, 10^o^, 15^o^) at which we know from previous experiments that F-activation is negligible [12]. In this experiment (Figure 8), cells co-expressing HPIV3 F and T193A HPIV3 HN were allowed to bind erythrocytes (RBCs) at 4°C. The cells were then washed and medium containing VIKI-PEG_4_-chol or untagged peptide (at a concentration of 10 uM), or no peptide, was added. The cells were transferred to the indicated temperatures to allow F-activation to occur, and after 60 minutes, zanamivir was added to interfere with HN-receptor interaction [7], [8], [9], [10], [58], [59]. We then determined the amount of target RBCs that were either released into the medium, irreversibly bound (not released by zanamivir), or fused, as for the experiment in Figure 6. Figure 8 shows the % of RBCs that were irreversibly bound at each temperature, in the presence of VIKI-PEG_4_-chol (red bars), untagged VIKI peptide (green bars) or no peptide (blue bars). In the absence of peptide, at every temperature all or almost all of the RBCs were either fused or released by zanamivir, indicating a lack of fusion peptide insertion into the target membrane. In the presence of VIKI-PEG_4_-chol however, a remarkable 20% of RBCs are irreversibly bound at 10^o^, a temperature too cold for fusion peptide insertion [12], [46]. At 15^o^, 65% of RBCs are irreversibly bound in the presence of VIKI-PEG_4_-chol. At this temperature, 20% of RBCs are irreversibly bound in the presence of the untagged VIKI peptide, corresponding to the extent of inhibition by the untagged peptide after fusion peptide insertion into the target membrane. The difference between the 65% retention by VIKI-PEG_4_-chol and the 20% retention by the corresponding untagged peptide represents the portion of the RBCs that are retained by the tagged peptide prior to fusion peptide insertion. At 25^o^, both peptides inhibit fusion to the same degree, and the experiment can no longer distinguish between peptide interaction that occurs before or after fusion protein insertion.

**Figure 9:**
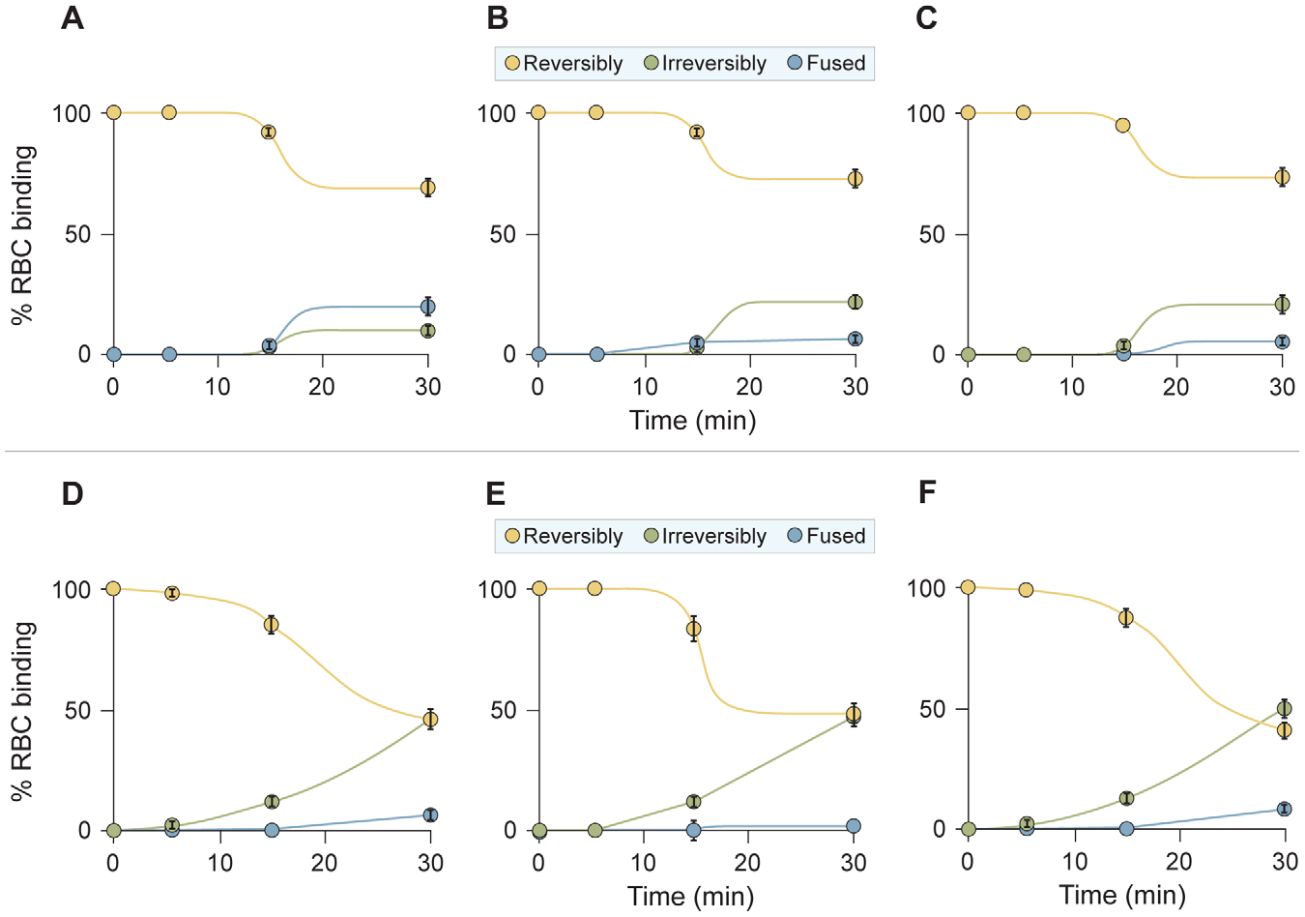
HPIV3 F HRC cholesterol-tagged peptides capture fusion intermediates of native F before fusion peptide insertion in the target membrane. Monolayers of cells co-expressing HN and F were allowed to bind to receptor-bearing RBCs at 4°C. Upon transfer to 15°C, media containing (A) no peptide, (B) 1 uM untagged peptide, (C) 1 uM VIKI-untagged peptide, (D) 1 uM V-chol peptide, (E) 1 uM V-PEG_4_-chol peptide, or (F) 1 uM VIKI-PEG_4_-chol peptide were added. Zanamivir was added to block HN-receptor interaction at the indicated timepoints and RBCs that were reversibly bound by HN-receptor interaction (yellow), irreversibly bound (green), or fused (blue) were quantified. The ordinate values are means (± SD) of results from triplicate samples.

Since the experiment at 15^o^ is most indicative of the interaction of the cholesterol-tagged peptide with native F protein at an early stage of activation, we carried out a time course experiment at this temperature (Figure 9). In this experiment, cells co-expressing wild-type cleaved F and HN were allowed to bind RBCs at 4°C. The cells were then washed and medium containing V-chol, V-PEG_4_-chol, VIKI-PEG_4_-chol, V-untagged, VIKI-untagged (at a concentration of 1uM), or no peptide, was added. The cells were transferred to 15°C, and at the indicated time points zanamivir was added. As expected, only minimal fusion was mediated at this temperature at 5 or 15 minutes. In the absence of peptide (panel A), most of the RBCs were released by zanamivir, indicating that binding is completely HN-dependent at 5, 15 and 30 minutes. In the presence of untagged peptides (panels B, C), the level of irreversibly bound peptide correlates directly with the onset of fusion, and represents inhibition of the progress of fusion once fusion peptide insertion occurs. However, for all the cholesterol tagged peptides (D, E, F), there is a progressive increase in the quantity of RBCs that are not detached by zanamivir, and are thus irreversibly bound, at times before fusion peptide insertion (15 and 30 minutes, compare to untagged peptide panels B, C). At 30 minutes, ∼50% of RBCs are irreversibly bound in the presence of chol-tagged peptides, although at this time point only ∼20% have inserted their fusion peptide (i.e., retained by untagged peptides.) This result indicates that in the presence of the cholesterol-tagged peptides, RBCs are retained in an HN-independent manner, with the peptide-F interaction causing the RBCs to remain attached to the HN/F-expressing cells at a stage before fusion peptide insertion.

Overall, these results indicate that the C-terminally cholesterol-tagged peptides can interact with F before the insertion of the fusion peptide into the membrane, that the sequence-optimized VIKI-PEG_4_-chol interacts better with this early stage of F, and that this interaction is the basis for the peptide's efficacy.

### The sequence-optimized, cholesterol-tagged peptide penetrates into the Central Nervous System

**Figure 10:**
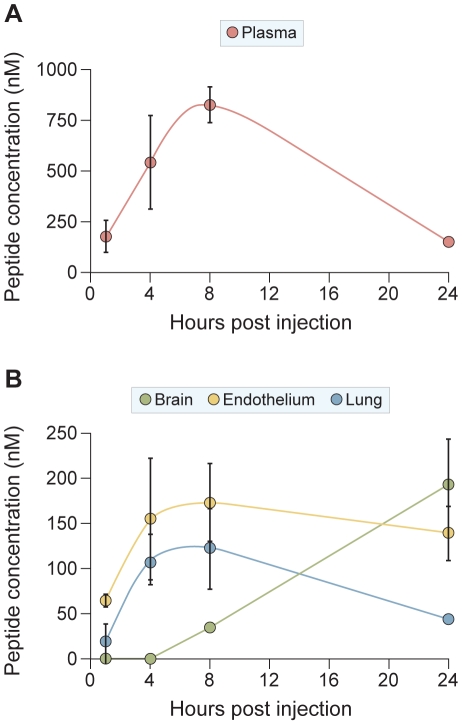
Biodistribution of sequence-optimized, cholesterol-tagged peptide: Central Nervous System penetration. Golden hamsters were injected with 2 mg/kg of VIKI -PEG_4_-chol peptide subcutaneously (s.q.) At indicated times (x-axis) the animals (three for each data point) were sacrificed, and the peptide concentrations in plasma (panel A), or in lung, endothelium and brain (panel B) were quantitated. The ordinate values are means (± SD) of results from three animals.

As a preliminary step towards testing VIKI-PEG_4_-chol in vivo, we assessed its pharmacokinetics (PK) in cotton rats, a model for HPIV3 infection [53], [60], and golden hamsters, a model for NiV infection [61], [62], [63]. We found that the peptide half-life is lower in cotton rats than in golden hamsters, and we therefore conducted further studies in golden hamsters. We specifically sought to determine whether the peptide biodistribution extends to the CNS — the target tissue of NiV. The biodistribution of VIKI-PEG_4_-chol in golden hamsters after a single subcutaneous injection of 2 mg/kg is shown in Figure 10. We observed an increase of the plasma peptide concentration to a peak of 800 nM after 8 hours (Figure 10A). Importantly, the plasma concentration after 24 hours was still approximately 200 nM, i.e. 100-fold the *in vitro* IC_50_ value for NiV [43] (see also Table 3). This indicated that a single daily dose of peptide would be enough to maintain a therapeutic dosage *in vivo*.

We found high concentrations of peptide also in the lung, endothelium, and brain (Figure 10B), the organs that are particularly relevant in the course of NiV infection. The peptide concentration in the lung and endothelium peaks at 8 h, with values of 120 nM and 170 nM, respectively, and slowly falls to 40 nM and 130 nM at 24 hours, values still considerably higher than the *in vitro* IC_50_. As expected, the kinetics of accumulation of the peptide in the brain is shifted with respect to the peak of plasma concentration. The peptide is first detected at 8 h (34 nM) and its brain concentration increases to 190 nM after 24 h.

### The sequence-optimized, cholesterol-tagged peptide is efficacious in vivo

**Figure 11:**
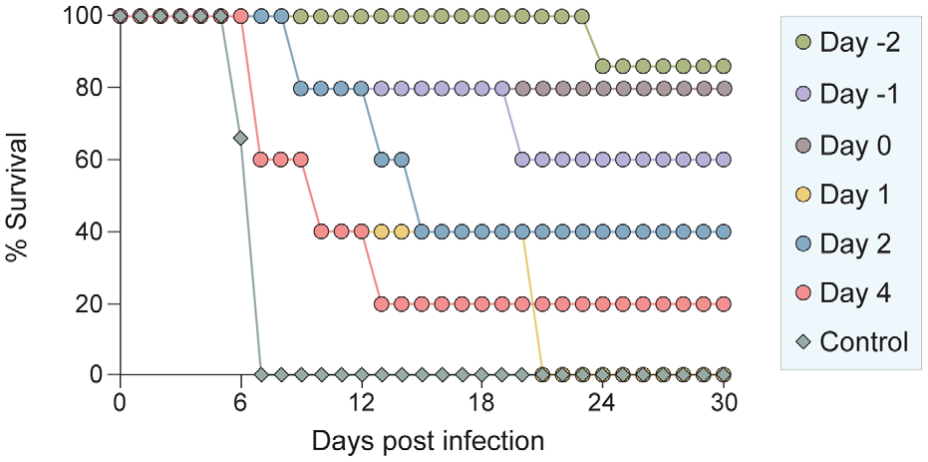
*In vivo* efficacy of sequence-optimized, cholesterol-tagged peptide. VIKI -PEG_4_-chol peptide injections were given to groups of 5 hamsters at 1 or 2 days before infection, concurrently with live NiV infection (day 0), and at 1, 2 or 4 days after infection. Injections were then repeated every day for up to 14 days post infection. Control animals were injected with vehicle alone.

We next evaluated the prophylactic and therapeutic efficacy of VIKI-PEG_4_-chol. The peptide was administered to golden hamsters intraperitoneally, either concurrently with virus infection, or with targeted post-exposure and prophylactic therapeutic regimes. Based on the biodistribution study, the peptide dose was 2 mg/kg administered daily, to achieve a plasma concentration ≥100x the in vitro IC_50_ throughout the 24 hours. Peptide injections were administered to groups of 5 hamsters at 2 days or 1 day before infection, concurrently with infection, and at 1, 2 or 4 days after infection. Injections were then repeated every day for up to 14 days post-infection. The hamsters were challenged by intraperitoneal inoculation of NiV, using 100x the LD_50_. The infected hamsters were observed daily for clinical signs (prostration, neurological signs) and variations in temperature and weight. All the untreated animals were euthanized within 7 days after infection due to neurological disease (Figure 11).

Treatment of animals 2 days before challenge resulted in 100% protection against disease for at least 21 days after infection (Figure 11). One of the surviving animals died at 23 days after infection (i.e. after 9 days without treatment) with neurological disease of unknown cause. Treatment concurrent with infection led to an 80% survival rate. Surprisingly, in light of the highly lethal nature of this virus, even treatment 2 days after infection led to survival of 40% of the animals. Of the animals treated 4 days post-infection, the onset of the illness was greatly delayed with a significantly extended lifespan, though only one of five animals survived to 30 days. While some of the findings need to be clarified with seroconversion and pathology studies, the overall picture is clearly one of protection and effective therapy for this otherwise lethal disease.

## Discussion

Fusion between viral and cell membranes initiates infection and is a key early step in the entry of enveloped viruses [3], [44]. Peptides derived from a number of viral fusion (F) proteins can inhibit infection. For the paramyxoviruses in analogy to HIV, F-derived peptides have been thought to interact with the fusion protein intermediate that exists after activation of the pre-cleaved F protein and the insertion of the fusion peptide into the target cell: this extended intermediate bridges the viral and cell membranes, and its refolding into a 6HB is a key driver for membrane fusion [45]. Peptides that correspond to the HRC region of F are believed to bind HRN domain of the extended intermediate and prevent its refolding into a the 6HB. The data reported in this manuscript however suggest that if HRC peptides are localized to the cell membrane by a cholesterol tag, and are engineered to interact more strongly with the HRN target domain, they become capable of interacting with F prior to the insertion of the fusion peptide into the target cell membrane. These peptides thus capture an earlier stage in the F-activation process.

### Which intermediate is the target of the sequence-optimized, cholesterol tagged peptides?

It is generally assumed that in order to mediate fusion and viral infectivity, the precursor (F0) protein must be cleaved into subunits F1 and F2 during transit to the cell surface, with the fusion peptide present at the F1 terminus but protected in the molecule's interior until activation. Our data however indicate that uncleaved F0 – only upon receptor engagement of the receptor binding protein, HN — undergoes conformational changes sufficient to expose the HRN domain to inhibitors. The interaction between uncleaved F0 and cholesterol-tagged peptide inhibitors is sufficient to irreversibly connect the viral and target cell membranes. We show this interaction both for a F protein cleavage-site mutant, incapable of fusion protein insertion, and for native, cleaved F protein at low temperature.

While our results provide the first biological evidence for conformational flexibility of an uncleaved precursor paramyxovirus fusion protein, the reported crystal structure of the uncleaved HPIV3 F appeared to be in a post-fusion state [57], suggesting that possibly the uncleaved precursor had undergone activation. Interestingly, similar conformational changes have been suggested for the uncleaved precursor of the influenza precursor fusion protein (HA_0_), which could be triggered to its irreversible pH-induced conformational change, without membrane insertion [64].

At 15^o^, all three cholesterol-tagged peptides bind to the early F intermediate and stabilize the pre-insertion state (Figure 9 and unpublished data). However at 37^o^, only the most efficient peptide is able to stabilize the intermediate. The efficiency with which the cholesterol-tagged inhibitors cause irreversible attachment is directly correlated to their inhibitory potency. The VIKI-PEG_4_-chol peptide, which interacts with the uncleaved, activated F protein most effectively, was designed to bind more avidly to the HRN, was shown by biophysical data to do so, and is the most effective inhibitor of infection. It is therefore tempting to speculate a causal connection between targeting an earlier intermediate along the reaction coordinate and increased potency, via an extended temporal window of opportunity. While more studies will be necessary to understand the mechanism of action, it is already clear at this stage that our design strategy is highly effective in vivo, as shown in an animal model of lethal NiV infection.

NiV has emerged as an important new cause of fatal encephalitis in humans. While the virus targets both the vascular endothelium and neurons, human disease is dominated by severe encephalitis in the acute phase, with severe brainstem involvement in fatal cases [65]. Even after recovery from acute encephalitis, NiV may persist in the central nervous system. During the late-onset encephalitis form of disease, the virus is exclusively localized to neurons.

For the henipaviruses, small animal models have been under development in recent years. While tropism for the endothelium is a hallmark of infection in both humans and animals, the various animal models have different degrees of CNS involvement. The animal model used in this study, the golden hamster, develops CNS manifestations of disease similar to the human disease, while other animal models, for example the cat [66], the ferret [67] or the naturally infected pig [68] develop primarily respiratory disease [61], [66], [67], [69]. We contend that the CNS component is essential to assess therapies that may be useful in human disease.

Several recent studies have raised the possibility of active or passive immunoprotection for henipaviruses [62], [63], [70]. In the cat, a subunit vaccine based on soluble glycoproteins protected the animals from subsequent viral infection [66]. While active immunization may be a useful strategy for livestock, it is unlikely that a broad human vaccine campaign for these pathogens is feasible. Recently, in ferrets, passive immunotherapy with a human monoclonal antibody produced partial protection when a high dose of the antibody was injected IV at 10 hours post infection with NiV [67]. However, in ferrets the NiV disease is predominantly respiratory, with a neurological syndrome observed only occasionally (2), and therefore to be effective in ferrets, a therapy may not need to reach the CNS.

The peptide reported here localized effectively in the CNS, and may offer protection in protected sites that are inaccessible to antibodies or hydrophilic small molecules. For example, although ribavirin inhibits NiV and HeV efficiently in vitro [71], it only slightly decreased the mortality of NiV encephalitis [72]; this may be explained by its failure to cross the blood-brain barrier. In support of this notion, hamsters with measles encephalitis died with ribavirin treatment if the route of injection was intraperitoneal, while intracranial injection of ribavirin resulted in complete protection [73]. Recently, two new nonhuman primate models susceptible to Nipah virus infection have been described: Squirrel Monkeys [74] and African Green Monkeys (13). Infection in these primates is followed by the high death rates associated with acute neurological and respiratory illness, closely resembling the disease in humans. We plan to validate our candidate therapeutics in these models as a key for further clinical development [74], [75]. It should also be noted that the peptides described in the current work inhibit the virus at a post-attachment stage, and are therefore expected to act synergistically with agents, like the above described antibody [67], that block viral attachment. Combination therapy is now the mainstay for HIV and other viral diseases where multiple therapeutic options are available.

While our animal studies focused on NiV, this antiviral strategy is applicable to other enveloped viruses as well, including several important pathogens. For example, the fact that the peptides are equally effective on NiV and HeV in vitro (not surprising since the HRN regions of the two viruses are identical) suggests that HeV infection will also respond to these peptides in vivo. Moreover, based upon our data indicating efficacy of VIKI-PEG_4_-chol against the HPIV3 in the HAE model, we anticipate this approach may lead to effective prevention and treatment of HPIV. Together with respiratory syncytial virus (RSV) HPIV has a huge impact on illness and hospitalization of young infants worldwide. No drugs or vaccines are available for these two viruses, and development of antiviral drugs has been a great challenge.

From the point of view of inhibitor design, we anticipate several directions to further develop our HPIV3/henipavirus inhibitor. First, the major effect that we observed from an apparently small change — the introduction of a 4-unit PEG spacer between the C-terminal cysteine of the peptide and the cholesterol moiety — indicates that there is considerable structural space to explore by varying the type and length of the linker. The optimal structure will have to strike the appropriate balance between solubility, pharmacokinetics, and potency. Second, the demonstration that increases in the *k_on_* of the inhibitor driven by the cholesterol moiety can be successfully combined with decreased inhibitor *k_off_* driven by improved protein-protein interactions, opens the possibility to independently optimize the two parameters and then combine them, as we did for the VIKI mutant. Future studies will also address the mechanism whereby the cholesterol tagged peptide crosses the blood-brain barrier. This mechanism is currently unknown and of great interest, with implications for treatment of CNS diseases caused by enveloped viruses, for example the CNS manifestations of HIV infection [76]. Current peptide antivirals (e.g. enfuvirtide) fail to cross the blood-brain barrier [77], [78], and this has impaired the treatment of CNS diseases. Design of peptides that can cross into the CNS could provide better therapeutics.

Finally, since cholesterol tagging drives peptide insertion in the target cell membrane, it may be applicable also to viruses that fuse in the cell interior: the peptide may be carried into the cell along with the entering virus, and exert its inhibitory action intracellularly. Fusion inhibitors for such viruses, including influenza, have been viewed as unlikely because the influenza fusion protein (HA) acts only once it is sequestered in an intracellular compartment. However lipid- tagged peptides may accompany the HA from the cell surface to the site of fusion activation, and we have obtained preliminary evidence for this antiviral mechanism.

## Materials and Methods

### Chemicals

4-guanadino-DANA (zanamivir) (**I**) was prepared from Relenza Rotadisks (5 mg zanamivir with lactose). A 50 mM stock solution was prepared by dissolving each 5 mg blister capsule in 285 µL serum-free medium. Stock solutions were stored at −20°C.

### Plasmids and reagents

The genes of NiV wt G and wt F were codon optimized and synthesized by GeneArt (Germany) and subcloned into the mammalian expression vector pCAGGS using EcoRI or XhoI and BglII.

### Peptide synthesis

All peptides were produced by standard Fmoc-solid phase methods. The cholesterol moiety was attached to the peptide via chemoselective reaction between the thiol group of an extra cysteine residue, added C-terminally to the sequence, and a bromoacetyl derivative of cholesterol, as previously described [43], [51], [52].

### Protein expression and purification

Substitutions were introduced into the homotypic and chimeric N42(L6)C32 constructs [48] using the method of Kunkel [79] and verified by DNA sequencing. All recombinant proteins were expressed in *E*. *coli* BL21(DE3)/pLysS (Novagen). Cells were lysed by glacial acetic acid and centrifuged to separate the soluble fraction from inclusion bodies. The soluble fraction containing protein was subsequently dialyzed into 5% acetic acid overnight at 4°C. All proteins were purified to homogeneity by reverse-phase HPLC (Waters, Inc) on a Vydac C18 preparative column (Hesperia, CA) using a water–acetonitrile gradient in the presence of 0.1% trifluoroacetic acid and lyophilized. Protein identities were confirmed by electrospray mass spectrometry (PerSeptive Biosystems Voyager Elite, Cambridge, MA). Protein concentrations were determined by using the method of Edelhoch [80].

### Crystallization and structure determination

HPLC-purified N42_NiV/HeV_(L6)C32_HPIV3_ was crystallized at room temperature using the hanging-drop vapor diffusion method by equilibrating against reservoir buffer (35% t-butanol, 0.1 M sodium citrate, pH 5.6), a solution containing 1 ml of 15 mg/mL protein dissolved in water and 1 ml of reservoir buffer. Crystals belong to space group *P*2_1_2_1_2_1_ (a = 32.4 Å, b = 54.1 Å, c = 134.1 Å) and contain three monomers in the asymmetric unit, with a solvent content of 43.5% (Table 1). The crystals were harvested in 40% t-butanol, 0.1 M sodium citrate, pH 5.6, 15% glycerol and frozen in liquid nitrogen. Diffraction data were recorded at 100 K on a MAR M165 CCD detector at the beamline X4C of the National Synchrotron Light Source at Brookhaven National Laboratory. The images were indexed and integrated using a monoclinic unit cell with the program DENZO [81]. The intensities were scaled in *P*2_1_2_1_2_1_ symmetry with the program SCALEPACK [81]; the systematic absence of intensities indicates three 2-fold screw axes. Initial phases were determined by molecular replacement with the program Phaser [82] by using the structure of the monomeric HeV two-helix (PDB entry 1WP8) as a starting model. Three N42/C32 molecules were oriented and placed in the asymmetric unit with a Z score of 21.4 and a final refined LLG of 669. In order to remove model bias, this model and the dataset for N42_NiV/HeV_(L6)C32_HPIV3_ were directly fed to the program Arp/Warp [83], which allowed ∼81% of the final model to be automatically traced. The resulting electron density map was of excellent quality and showed the location of most of the side chains. The linker region of chain B was built into the evident electron density and the linker regions of chains A and C are not visible in the electron density maps and therefore must be disordered. Density interpretation and manual model building were done with the program O [84]. Crystallographic refinement of this model was carried out by using Refmac [85], resulting in the *R*_free_ of 29.2% and the *R*_work_ of 23.3% between 67.1 and 1.80 Å resolution. At this stage, one citrate ion, two t-butanol molecules and 156 water molecules were modeled in the electron density. Refinement was concluded using Refmac [85] with TLS groups assigned for each N42 or C32 monomer [86]. The final model (*R*_cryst_ = 19.4% and *R*_free_ = 24.2% for the resolution range 67.1–1.80 Å) consists of residues 3–42 and 48–80 (monomer A), 2–80 (monomer B) and 1–39 and 50–80 (monomer C), one citrate ion, two t-butanol molecules and 156 water molecules in the asymmetric unit. Bond lengths and bond angles of the model have rms deviations from ideality of 0.011 Å and 1.2°, respectively. The rms deviations were calculated with LSQKAB in CCP4i program suite [87].

### Circular dichroism spectroscopy

CD experiments were performed on an Aviv 62A/DS (Aviv Associates, Lakewood, NJ) spectropolarimeter equipped with a thermoelectric temperature control in TBS (50 mM Tris-HCl, pH 8.0, 150 mM NaCl) and 50 uM protein. CD spectra were collected from 260 to 200 nm at 4°C by using an average time of 5 s, a cell pathlength of 0.1 cm and a bandwidth of 1 nm. A **[** *theta* **]_222_** value of –35,000 deg cm^2^ dmol^−1^ was taken to correspond to 100% helix [88]. Thermal stability was determined by monitoring **[** *theta* **]_222_** as a function of temperature in TBS (pH 8.0) and with the addition of 3 or 5 M guanidine hydrochloride (GuHCl) to facilitate unfolding. Thermal melts were performed in two-degree intervals with a 2-min equilibration at the desired temperature and an integration time of 30 s. Reversibility was verified by repeated scans. Superimposable folding and unfolding curves were observed, and >85% of the signal was regained upon cooling. Values of midpoint unfolding transitions (*T*_m_) were estimated by evaluating the maximum of the first derivative of [*q*]_222_ *versus* temperature data [89].

### Sedimentation equilibrium analysis

Analytical ultracentrifugation measurements were carried out on a Beckman XL-A (Beckman Coulter) analytical ultracentrifuge equipped with an An-60 Ti rotor (Beckman Coulter) at 20°C. Protein samples were dialyzed overnight against TBS (pH 8.0), loaded at initial concentrations of 30, 90 and 300 uM, and analyzed at rotor speeds of 19 and 22 krpm. Data were acquired at two wavelengths per rotor speed setting and processed simultaneously with a nonlinear least squares fitting routine [90]. Solvent density and protein partial specific volume were calculated according to solvent and protein composition, respectively [91]. Apparent molecular masses were all within 10% of those calculated for an ideal trimer, with no systematic deviation of the residuals.

### Transient expression of G and F genes

Transfections were performed according to the Lipofectamine 2000 manufacturer's protocols (Invitrogen).

### Cells and viruses

293T (human kidney epithelial), Vero (African green monkey kidney cells) and CV1 cells were grown in Dulbecco's modified Eagle's medium (DMEM) (Mediatech; Cellgro) supplemented with 10% fetal bovine serum and antibiotics in 5% CO_2_. The effect of peptides on HPIV3 plaque number was assessed by a plaque reduction assay performed as described previously [92]. Briefly, CV-1 cell monolayers were inoculated with 100–200 PFU of HPIV3 in the presence of various concentrations of peptides. After 90 minutes, 2X minimal essential medium containing 10% FBS was mixed with 1% methylcellulose (or avicell) and added to the dishes. The plates were then incubated at 37°C for 24 hours. After removing the medium overlay, the cells were immunostained for plaque detection. The number of plaques in the control (no peptide, scrambled peptide) and experimental wells were counted under a dissecting stereoscope.

### Human airway epithelial (HAE) cultures

The EpiAirway AIR-100 system (MatTek Corporation) consists of normal human-derived tracheo/bronchial epithelial cells that have been cultured to form a pseudo-stratified, highly differentiated mucociliary epithelium closely resembling that of epithelial tissue in vivo. Upon receipt from the manufacturer, HAE cultures were transferred to 6-well plates (containing 0.9 ml medium per well) — with the apical surface remaining exposed to air — and incubated at 37°C in 5% CO_2_ overnight.

### Infection and treatment of HAE and measurement of viral titers from infected HAE

HAE cultures were infected by applying 200 ul of EpiAirway medium containing 4000 pfu of wt HPIV3 to the apical surface for 90 min at 37°C. At 90 minutes, the medium containing the inoculum was removed, cultures were placed at 37°C and fed each day via the basolateral surface with 0.9 ml medium. Viruses were harvested by adding 200 ul medium per well to the HAE cultures' apical surface and allowed to equilibrate for 30 min at 37°C. The suspension was then collected and viral titers were determined as previously described [53]. This viral collection was performed sequentially on the same wells of cells on each day post-infection. Treatments were performed by adding medium containing 1 uM of the indicated peptide at the time of infection only. Harvesting by the apical surface washes was done sequentially on the same HAE cultures, at the indicated time points.

### Pseudotyped virus infection assay

The VSV-ΔG-RFP is a recombinant VSV derived from the cDNA of VSV Indiana, in which the G gene is replaced with the Ds-Red (RFP) gene. Pseudotypes with NiV G and F were generated as described [93], [94]. Briefly, 293T cells were transfected with plasmids encoding either NiV G/F. Twenty-four hours post-transfection, the dishes were washed and infected (m.o.i. of 1) with VSV-ΔG-RFP complemented with VSV G. Supernatant fluids containing pseudotyped virus NiV G/F were collected 24 hrs post-infection and stored at −80°C. For infection assays, NiV G/F pseudotyped viruses were used at an m.o.i. of 0.25 to infect 293T cells. Peptides were added at various concentrations. RFP production was analyzed by FACS (Benton Dickinson FACSCalibur) [42], [48].

### Beta-galactosidase complementation-based fusion assay

We adapted an assay [95] that detects early stages of fusion activation, since the read-out does not depend upon downstream transactivation events. We used this assay for experiments in which the greater range of detection of this assay was necessary. The assay is based on alpha complementation of beta-gal; the beta-gal protein lacking the N-terminal 85 residues (omega peptide) is expressed from one plasmid, and the N-terminal 85 residues (alpha peptide) is expressed from a second plasmid. Cell fusion leads to complementation, and beta-galactosidase is quantitated using the Galacto-Star (Applied Biosystems) chemiluminescent reporter gene assay system. Receptor bearing cells expressing the omega peptide are mixed with viral glycoproteins co-expressing cells that also express the alpha peptide, at various temperatures and at specific time points. Fusion is stopped by lysing the cells with lysis buffer.

### Assays for F-activation, receptor retention and receptor release

Monolayers of 293T cells transiently expressing F-csm and mutant HN T193A were washed three times and incubated with 1% RBC suspensions at pH 7.5 for 30 min at 4°C. After rinsing to remove unbound RBCs, 1uM of peptide was added, the plates were placed at the 37°C for the indicated time, zanamivir at a final concentration of 10 mM was added and the plates left at 37°C until the end of the latest time point (60minutes), plates then were rocked and the liquid phase was collected in V-bottomed tubes for measurement of released RBCs. The monolayers were then incubated at 4°C with 200 mL of RBC lysis solution; lysis of unfused RBCs with NH_4_Cl removes the RBCs whose membranes have not fused with HN/F co-expressing cells. The liquid phase was collected in V-bottomed 96-well plates for measurement of reversibly bound RBCs. The cells were then lysed in 200 µL 0.2% Triton X-100/PBS and were transferred to flat-bottom 96 well plates for quantification of the pool of fused RBCs. The amount of RBCs in each of the above three compartments was determined by measurement of absorption at 410 nm.

### Biodistribution analysis

15 male Syrian Golden hamsters were obtained from a commercial source and housed in an AAALAC-approved facility until dosing. The animals were dosed subcutaneously with 2 mg/kg of peptide dissolved in PBS. At the indicated time points, blood was collected by cardiac puncture into chilled, Na-heparin tubes, the plasma was separated by centrifugation, snap frozen, and stored at −80°C until analysis. Brain, lung, and endothelial (aorta and vena cava) tissues were also collected, homogenized in 1 ml of PBS per gram of tissue and frozen.

Analytical samples in plasma or tissue homogenate were prepared by precipitation of proteins with three volumes of acetonitrile and 10% formic acid. After centrifugation, the supernatant was analyzed by LC/MS/MS using a C18 reverse phase HPLC column and an acetonitrile-water gradient system. Peaks were analyzed by mass spectrometry (MS) using ESI ionization in MRM mode. A calibration curve was prepared from 50-fold dilution of PBS peptide solutions into plasma or tissue homogenate, as appropriate, processed as above.

### Ethics statement

All work with live Nipah virus was performed in the BSL-4 laboratory of the NIAID/Rocky Mountain Laboratories (RML). All research involving animals was conducted in strict accordance with Animal Care and Use guidelines of the National Institutes of Health. The protocol was approved by the Animal Care and Use Committee at RML (National Institutes of Health Animal Study Proposal #2009-52), approved at NIAID/RML on 5/28/2009 and at Weill Cornell on 9/14/2009. Animal work was performed by certified staff in an Association for Assessment and Accreditation of Laboratory Animal Care (AAALAC) approved facility, and all efforts were made to minimize suffering.

### Golden hamster infection

Female Syrian hamsters (6-week-old from Harlan Laboratories) were anesthetized by chamber induction (5 Litres/min 100% O_2_ and 3–5% isoflurane). Each hamster was intraperitoneally (IP) inoculated with 1000 LD_50_ of NiV in a 200 ul volume. For peptide treatment, groups of 5 hamsters were treated with 2 mg/kg peptide in a 100 ul volume by IP injection daily under isoflurane anesthesia as described above. Treatment started on days -2, 0, 2 or 4 post infection and were continued up to 14 days post infection. All animals were weighed daily and observed for clinical signs. Animals were euthanized when severe neurological symptoms were observed or when a >20% weight loss was determined. All animals were housed under sterile conditions using individually ventilated cages.

## Supporting Information

Figure S1 HPIV3 F HRC C-terminally cholesterol-tagged peptides interact with uncleaved F: the peptide-F interaction attaches HN/F-expressing cells to receptor-bearing target cells. Monolayers of cells co-expressing HN and uncleaved (cleavage site mutant, csm) F were allowed to bind to receptor-bearing RBCs at 4°C. Upon transfer to 37°C, media containing 1 uM of the indicated peptides were added. Zanamivir was added to block HN-receptor interaction, and the irreversibly bound RBCs were quantified. The ordinate values are means (± SD) of results from triplicate samples.
(4.13 MB TIF)

Figure S2 Receptor-bearing target cells bearing HPIV3 F HRC C-terminally cholesterol-tagged peptides capture HN/F-expressing cells via peptide-F interaction. Receptor-bearing RBCs or cells co-expressing HN and uncleaved (csm) F were incubated with the indicated peptides for 1 hour at RT, and washed. Binding between RBCs and HN/F-expressing cells was allowed at 4°C. Unbound RBCs were washed away and the cells transferred to 37°C for one hour. Zanamivir was added to block HN-receptor interaction, and the irreversibly bound RBCs were quantified. The ordinate values are means (± SD) of results from triplicate samples.
(7.34 MB TIF)

Table S1 Viral titer collected from the apical surface of HAE cells infected with wt HPIV3, in the presence of the indicated treatments.
(0.05 MB PDF)
