# Supplementary figures and images for: Inhibition of Nipah Virus Infection In Vivo: Targeting an Early Stage of Paramyxovirus Fusion Activation during Viral Entry
Source: PLoS Pathog. 2010 Oct 28;6(10):e1001168. doi: 10.1371/journal.ppat.1001168 (PMC2965769; doi:10.1371/journal.ppat.1001168)

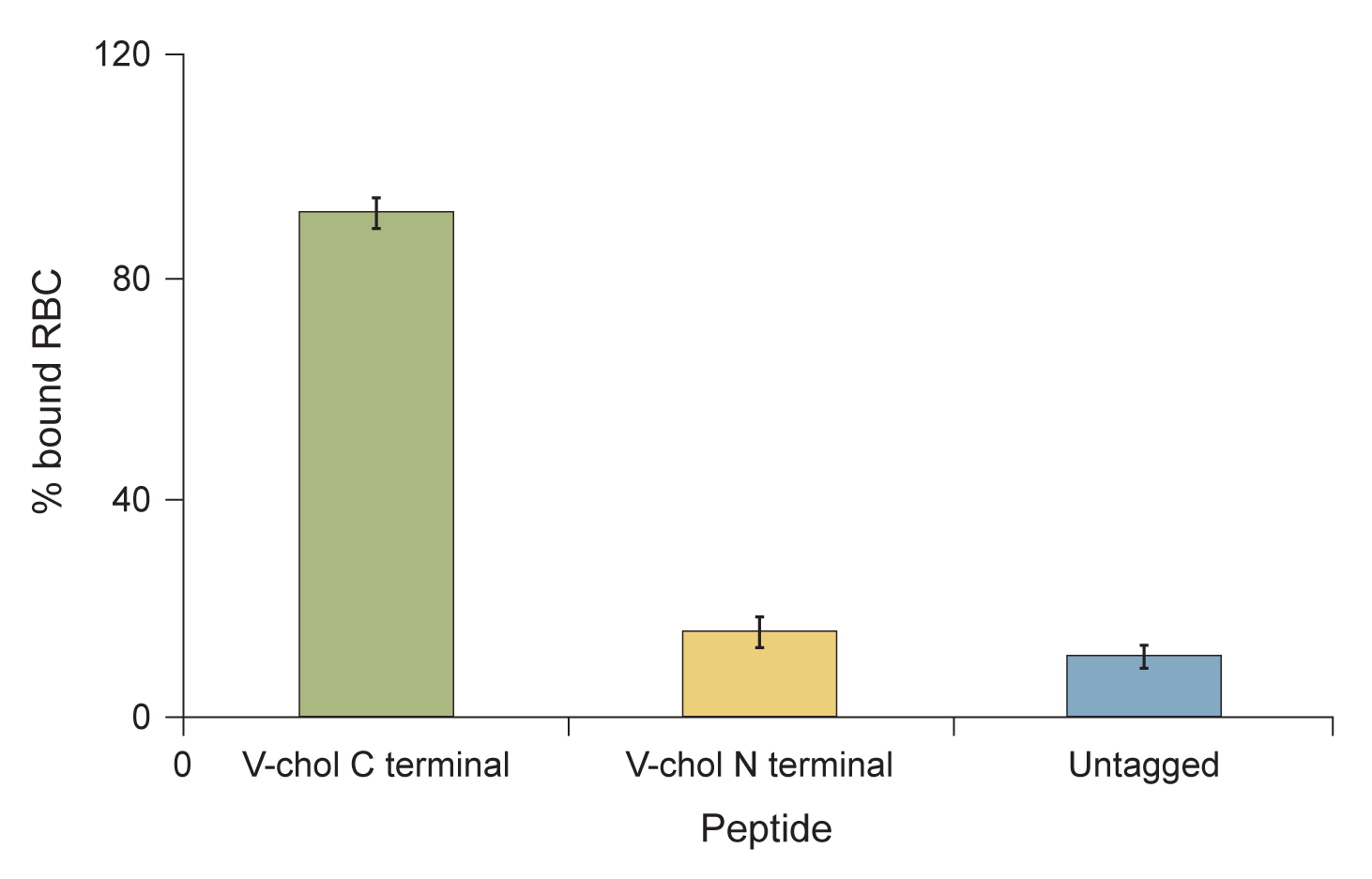

Supplement: Figure S1 — HPIV3 F HRC C-terminally cholesterol-tagged peptides interact with uncleaved F: the peptide-F interaction attaches HN/F-expressing cells to receptor-bearing target cells. Monolayers of cells co-expressing HN and uncleaved (cleavage site mutant, csm) F were allowed to bind to receptor-bearing RBCs at 4°C. Upon transfer to 37°C, media containing 1 uM of the indicated peptides were added. Zanamivir was added to block HN-receptor interaction, and the irreversibly bound RBCs were quantified. The ordinate values are means (± SD) of results from triplicate samples. (4.13 MB TIF) [file ppat.1001168.s001.tif]

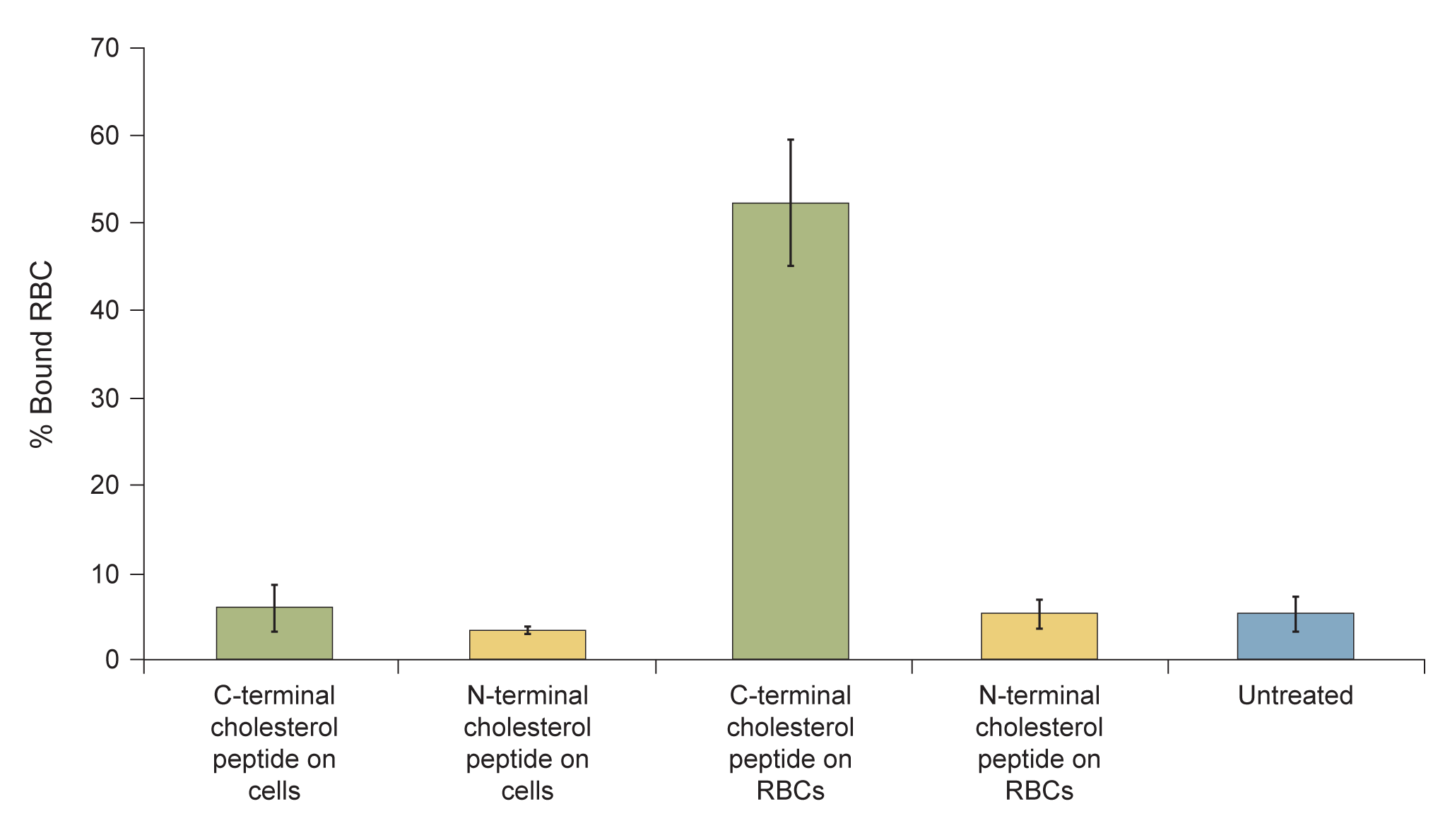

Supplement: Figure S2 — Receptor-bearing target cells bearing HPIV3 F HRC C-terminally cholesterol-tagged peptides capture HN/F-expressing cells via peptide-F interaction. Receptor-bearing RBCs or cells co-expressing HN and uncleaved (csm) F were incubated with the indicated peptides for 1 hour at RT, and washed. Binding between RBCs and HN/F-expressing cells was allowed at 4°C. Unbound RBCs were washed away and the cells transferred to 37°C for one hour. Zanamivir was added to block HN-receptor interaction, and the irreversibly bound RBCs were quantified. The ordinate values are means (± SD) of results from triplicate samples. (7.34 MB TIF) [file ppat.1001168.s002.tif]
