# Supplementary material for: Inhibition of Nipah Virus Infection In Vivo: Targeting an Early Stage of Paramyxovirus Fusion Activation during Viral Entry
Source: PLoS Pathog. 2010 Oct 28;6(10):e1001168. doi: 10.1371/journal.ppat.1001168 (PMC2965769; doi:10.1371/journal.ppat.1001168)
Supplement: Table S1 — Viral titer collected from the apical surface of HAE cells infected with wt HPIV3, in the presence of the indicated treatments. (0.05 MB PDF) [file ppat.1001168.s003.pdf]

| | HPIV3 viral titer (pfu/ml $10^3$) | | | | |
|---|---|---|---|---|---|
| | Day 1 | Day 2 | Day 3 | Day 5 | Day 7 |
| untreated | 0.6+/-0.2 | 3.8+/-0.9 | 7.4+/-0.6 | 4.6+/-1.8 | 1.0+/-0.4 |
| V-chol (1uM) | 1.0+/-0.8 | 3.8+/-0.9 | 8.5+/-2.7 | 12+/-3.0 | 2.7+/-0.8 |
| V-PEG$_4$-Chol (1uM) | 0.3+/-0.2 | 3.8+/-1.6 | 10+/-1.9 | 3.0+/-0.6 | 11+/-1.9 |
| VIKI-PEG$_4$-Chol (1uM) | 0.9+/-0.8 | 0.7+/-0.7 | 2.0+/-0.2 | 1.6+/-0.6 | 3.3+/-1.3 |

**Table S1.** Viral titer collected from the apical surface of HAE cells infected with wt HPIV3, in the presence of the indicated treatments.
